# Supplementary material for: Single nucleus RNA-sequencing defines unexpected diversity of cholinergic neuron types in the adult mouse spinal cord
Source: Nat Commun. 2021 Apr 30;12:2471. doi: 10.1038/s41467-021-22691-2 (PMC8087807; doi:10.1038/s41467-021-22691-2)
Supplement: Supplementary file 1 — Supplementary Information [file 41467_2021_22691_MOESM1_ESM.pdf]

Supplementary Figure 1

Wild-Type Control

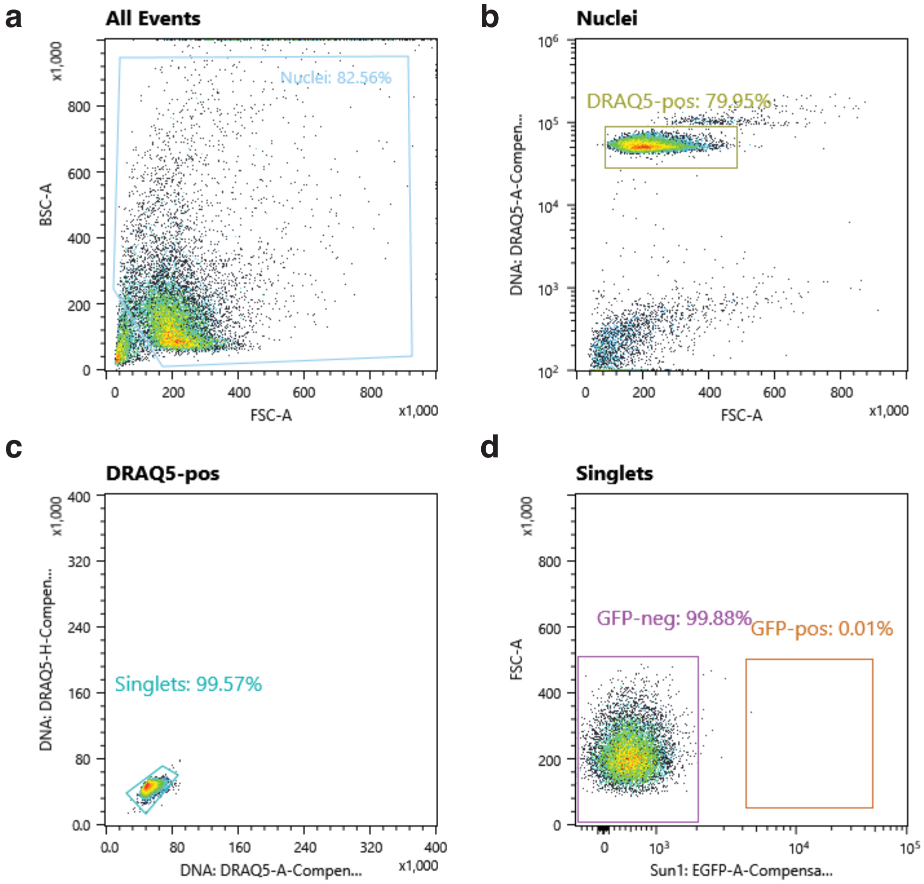

e Gates and Statistics

Name	Events	%Parent	%Total
All Events	15,517	0.00%	100.00%
Nuclei	12,811	82.56%	82.56%
DRAQ5-pos	10,242	79.95%	66.01%
Singlets	10,198	99.57%	65.72%
GFP-neg	10,186	99.88%	65.64%
GFP-pos	1	0.01%	0.01%

Chat$^{IRES\text{-}Cre/wt}$ x ROSA-Sun1sfGFP$^{+/wt}$

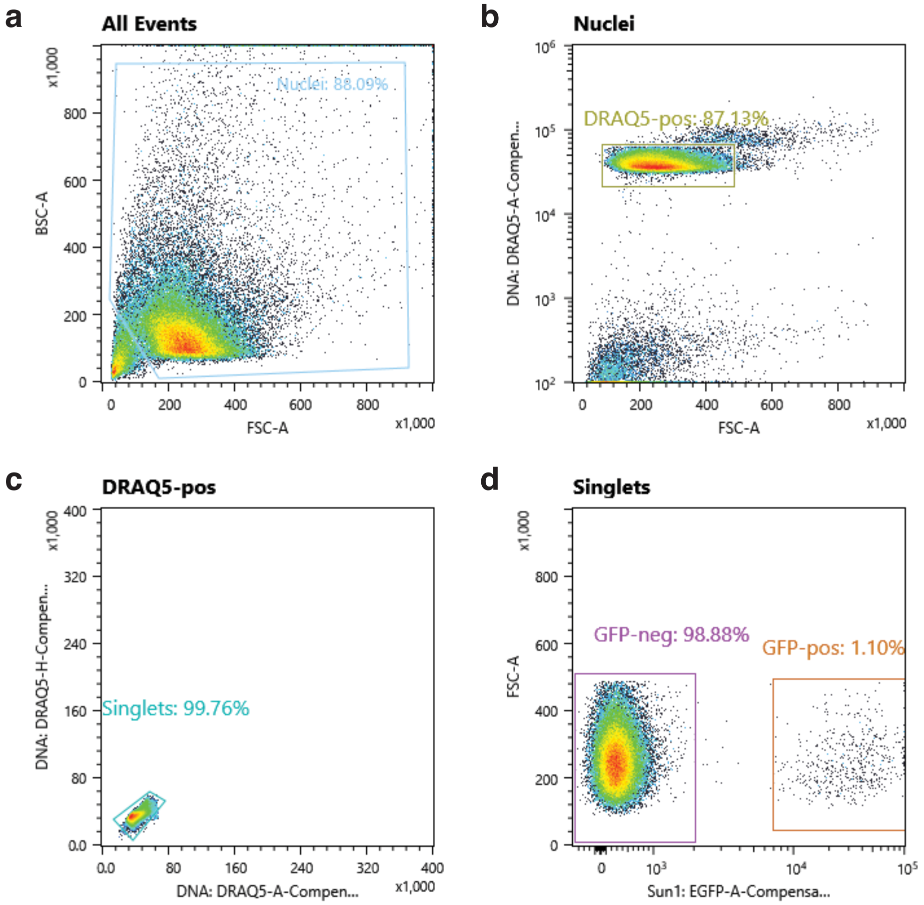

e Gates and Statistics

Name	Events	%Parent	%Total
All Events	65,304	0.00%	100.00%
Nuclei	57,524	88.09%	88.09%
DRAQ5-pos	50,119	87.13%	76.75%
Singlets	50,000	99.76%	76.56%
GFP-neg	49,440	98.88%	75.71%
GFP-pos	548	1.10%	0.84%

Supplementary Fig. 1. Quality control for selection of single GFP-positive nuclei by FACS sorting. Example FACS sorting summary from nuclear suspensions prepared from a wild type control sample (top), and a Chat-IRES-Cre::CAG-Sun1sfGFP sample (bottom). **a**, Nuclear suspension visualized by FACS, with removal of small debris via FSC vs BSC gate; **b**, selection of nuclei using DRAQ5 positive gate; **c**, removal of doublets using DRAQ5 height vs. area gate; **d**, collection of GFP+ nuclei; **e**, gating work flow and quantification of events in each gate, from All Events → Nuclei → Draq5-Pos → Singlets → GFP +/-.

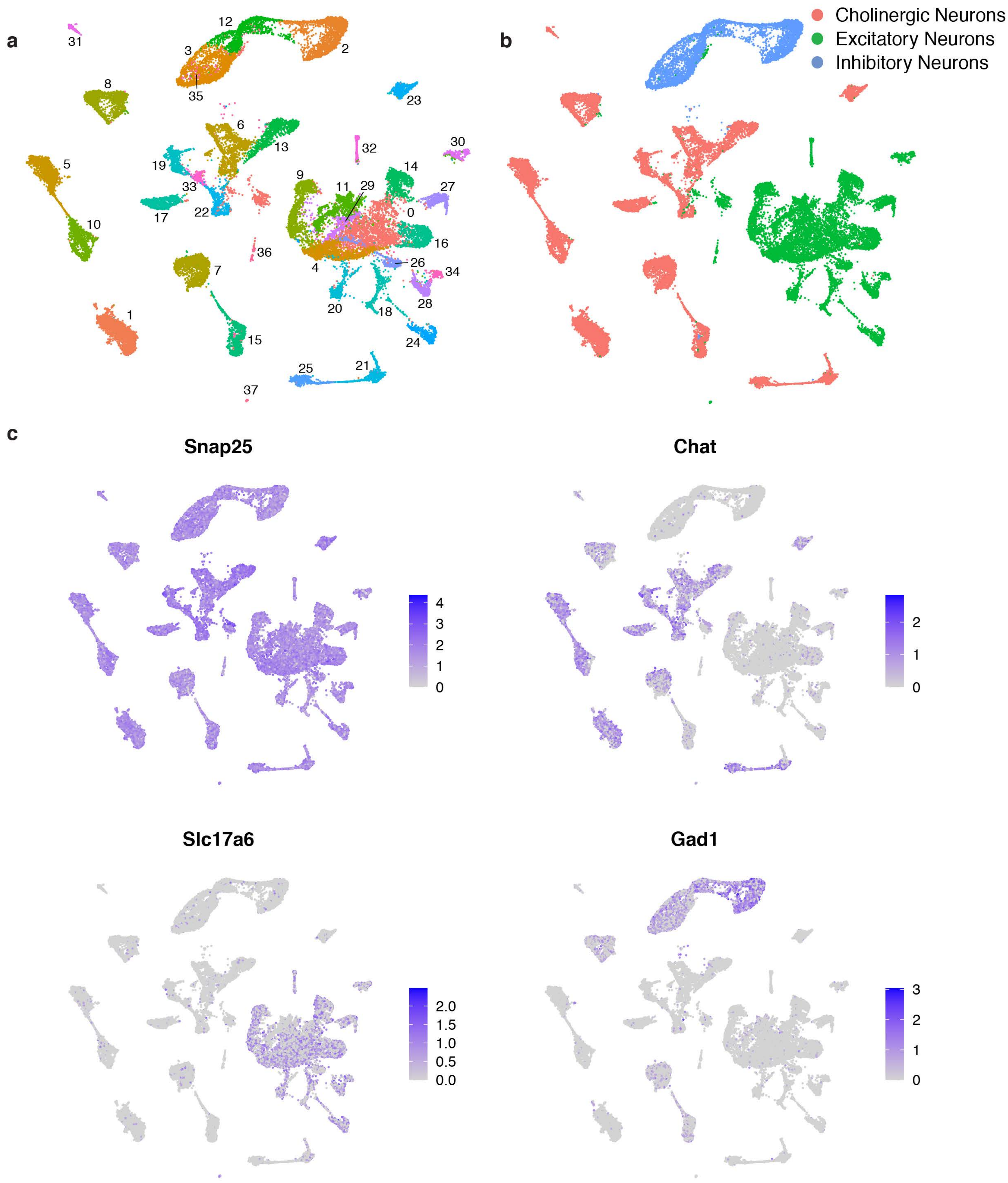

a
31
12
3
2
35
8
23
6
13
32
30
5
19
33
9
11
29
14
27
17
22
0
10
16
36
26
4
7
34
28
20
18
1
15
24
25
21
37
b
Cholinergic Neurons
Excitatory Neurons
Inhibitory Neurons
c
Snap25
4
3
2
1
0
Chat
2
1
0
Slc17a6
2.0
1.5
1.0
0.5
0.0
Gad1
3
2
1
0

Supplementary Fig. 2. Characterization of all nuclei sequenced and assignment of cholinergic clusters. a, UMAP showing all nuclei analyzed, segregated into 38 distinct clusters. **b**, UMAP showing assignment of clusters by cell type. **c,** Feature plots showing expression of the neuronal marker *Snap25*, the cholinergic neuron marker *Chat*, the excitatory neuron marker *Slc17a6*, and the inhibitory neuron marker *Gad1* across all nuclei analyzed in this study.

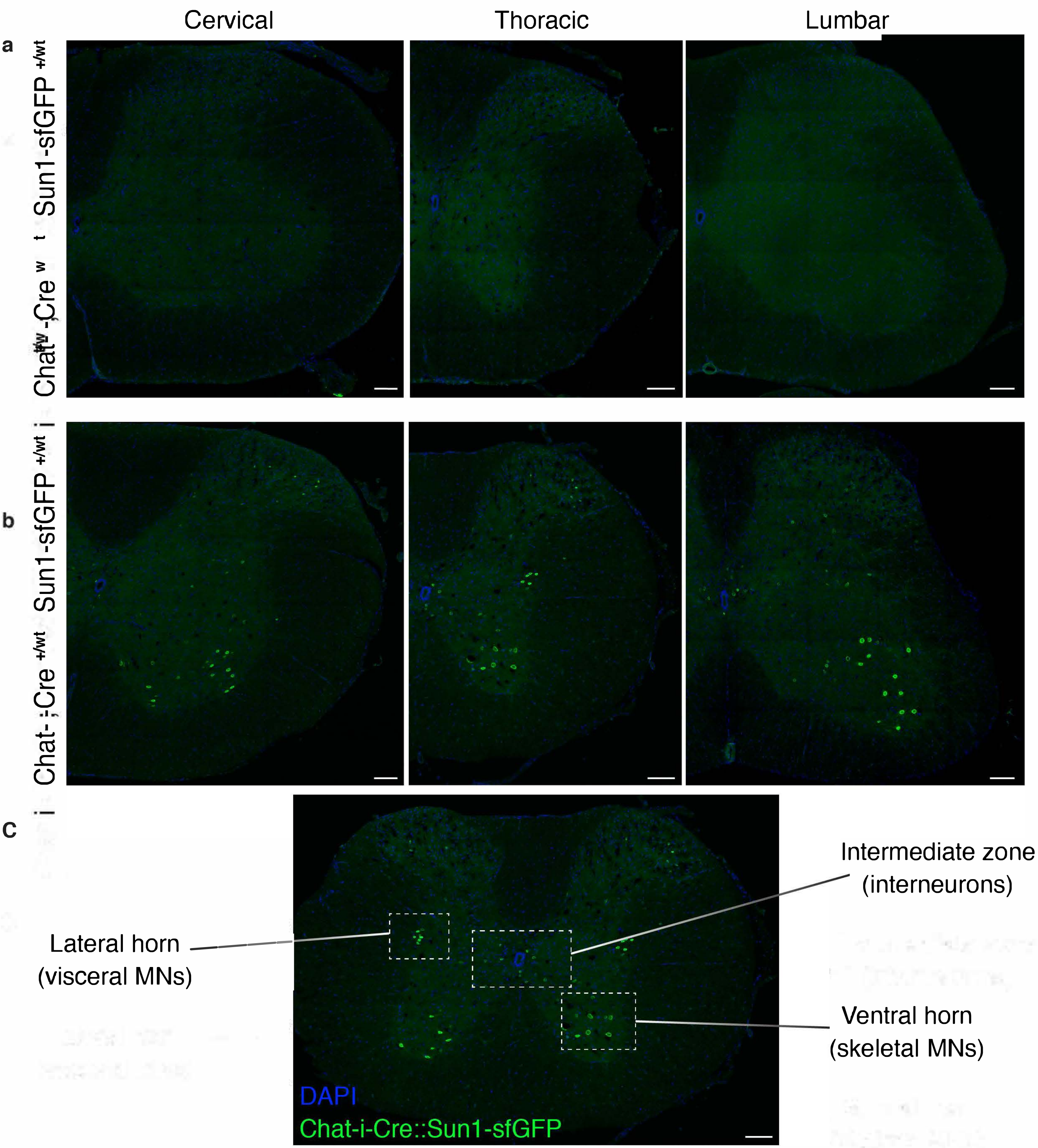

Supplementary Fig 3. Cre-negative mice do not express Cre-dependent Sun1-sfGFP. a, Examples of cervical, thoracic, and lumbar levels in Chat-Cre wt/wt; Sun1-sfGFP +/wt mice showing lack of baseline recombination events resulting in non-specific GFP expression. **b,** Examples of Chat-Cre +/wt; Sun1-sfGFP +/wt mice showing GFP expression. **c,** Example image highlighting the endogenously labeled visceral MNs in the lateral horn, skeletal MNs in the ventral horn, and interneurons in the intermediate zone. Scale bars, 100 μm.

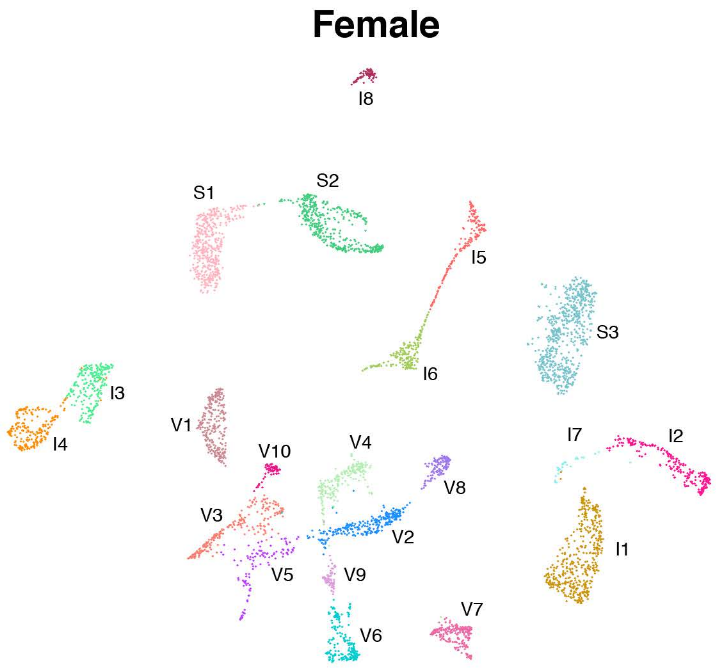

Supplementary Fig 4. Cholinergic neurons do not exhibit notable transcriptional sex differences. a, UMAPs showing female nuclei (left) and male nuclei (right) in each cluster. No cluster is unique to either sex.

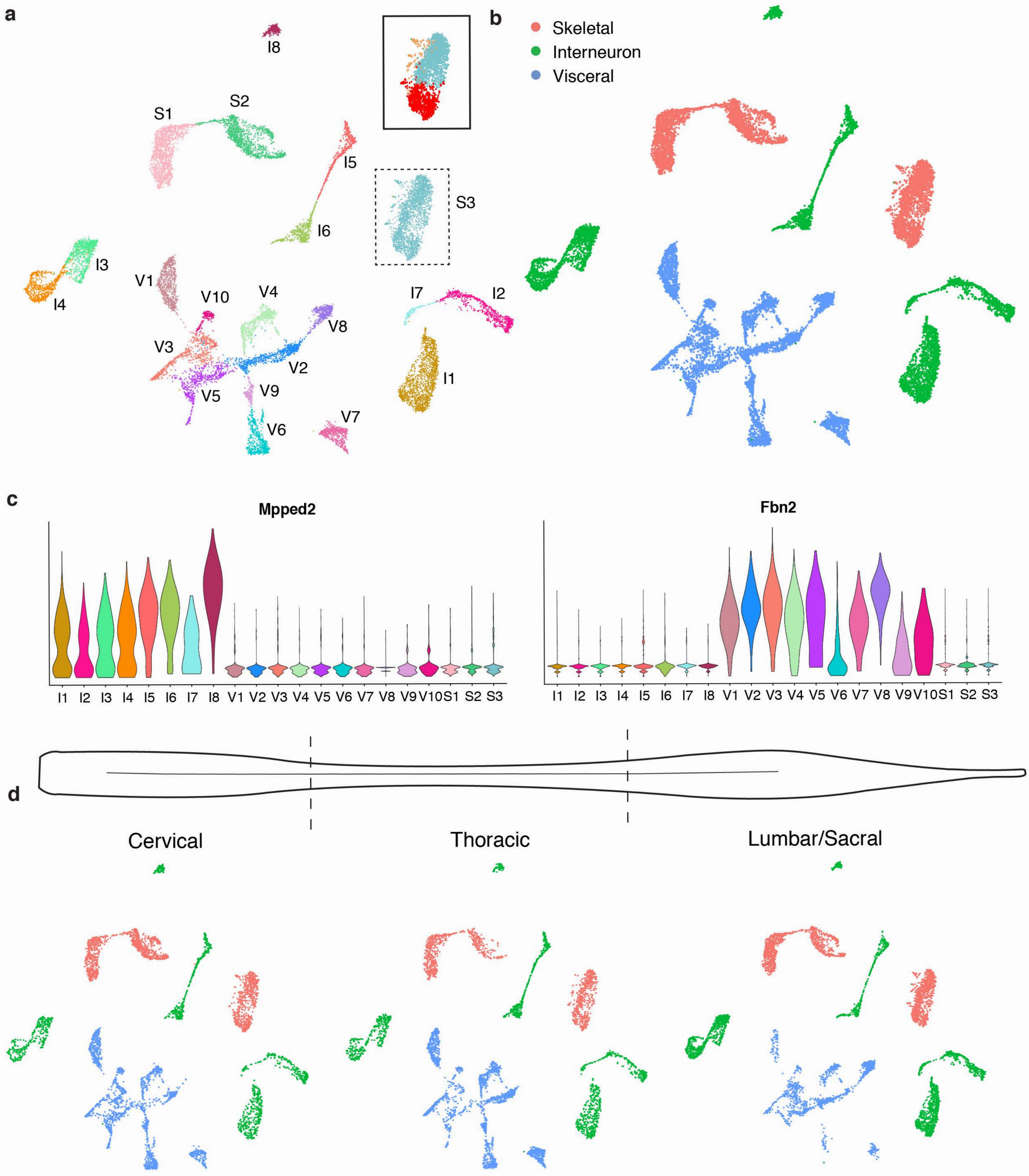
a
I8
S1
S2
I5
I6
S3
I3
I4
V1
V10
V4
V8
V3
V2
V5
V9
V6
V7
I7
I2
I1
b
Skeletal
Interneuron
Visceral
c
Mpped2
Fbn2
I1 I2 I3 I4 I5 I6 I7 I8 V1 V2 V3 V4 V5 V6 V7 V8 V9 V10 S1 S2 S3
d
Cervical
Thoracic
Lumbar/Sacral

Supplementary Fig. 5. Cholinergic neuron clusters can be classified by type and vary across spinal cord regions. a, UMAP of all cholinergic clusters. Prior to renaming, cluster S3 divided into 3 closely related groups (boxed) that were merged due to their high transcriptional similarity. **b,** UMAP showing skeletal (red), interneuron (green), and visceral (blue) clusters. **c**, *Mpped2* is a distinct marker for all cholinergic interneuron clusters, while *Fbn2* labels clusters V7 and V8 as visceral MNs. **d**, UMAPs of cells detected in each spinal cord region (cervical, thoracic, and lumbar/sacral) showing variability in cholinergic neurons along the rostro-caudal axis of the spinal cord.

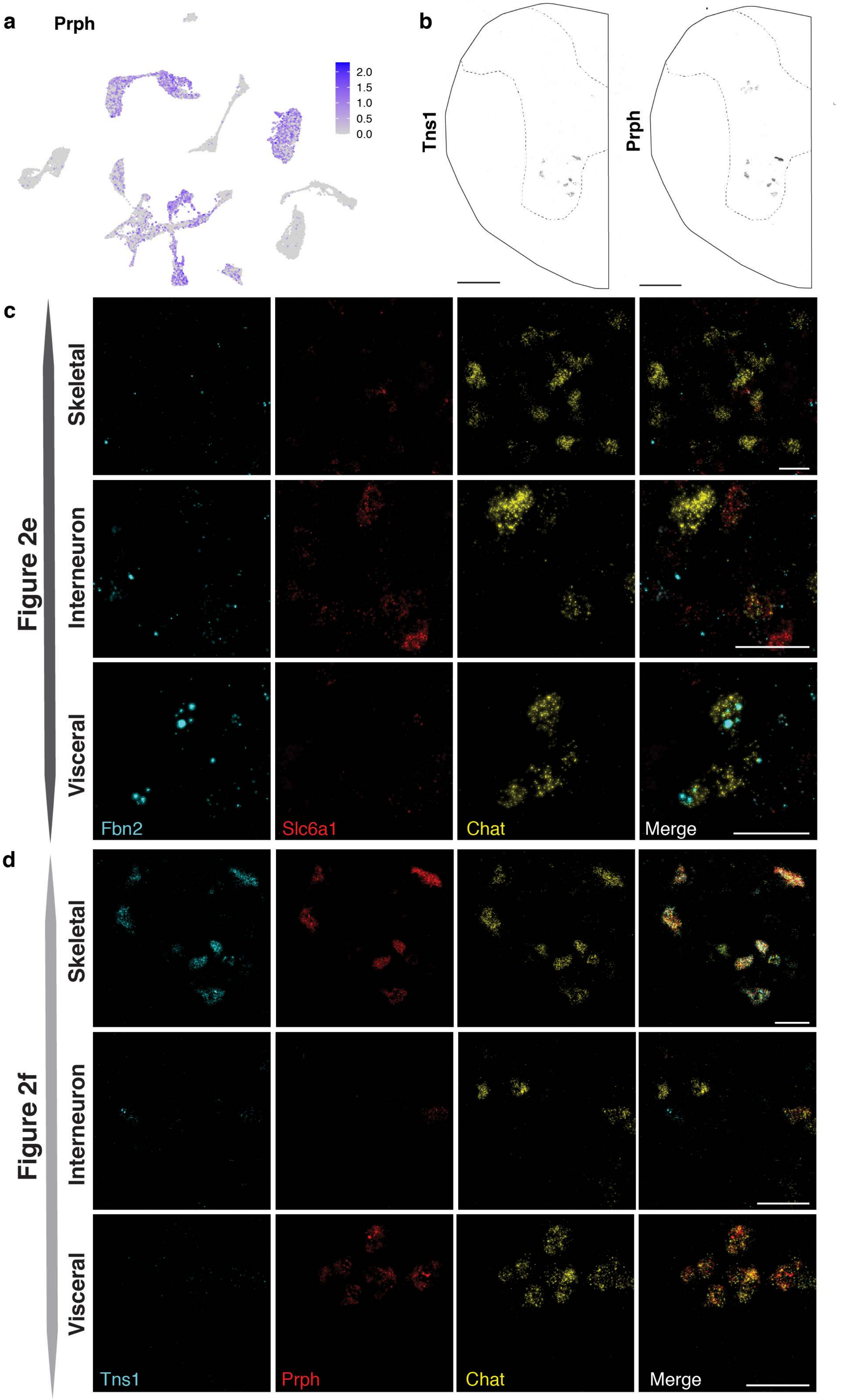

a
Prph
2.0
1.5
1.0
0.5
0.0
b
Tns1
Prph
c
Figure 2e
Skeletal
Interneuron
Visceral
Fbn2
Slc6a1
Chat
Merge
d
Figure 2f
Skeletal
Interneuron
Visceral
Tns1
Prph
Chat
Merge

Supplementary Fig. 6. Expression of cholinergic subtype markers. a, A feature plot showing extent of expression of *Prph* in our dataset across all cholinergic clusters. **b**, Low magnification images of *Tns1* expression, localized to the ventral horn, and *Prph* in ventral and lateral horn of thoracic spinal cord. **c**, High magnification images of skeletal MNs (ventral horn), interneurons (intermediate zone), and visceral MNs (lateral horn) from Figure 2e showing expression of *Fbn2* (cyan), *Slc6a1* (red), and *Chat* (yellow) in each channel individually, and merged. **d**, High magnification images of skeletal MNs (ventral horn), interneurons (intermediate zone), and visceral MNs (lateral horn) from Figure 2f showing expression of *Tns1* (cyan), *Prph* (red), and *Chat* (yellow) in each channel individually, and merged. Low magnification scale bars, 200 μm. High magnification scale bars, 50 μm.

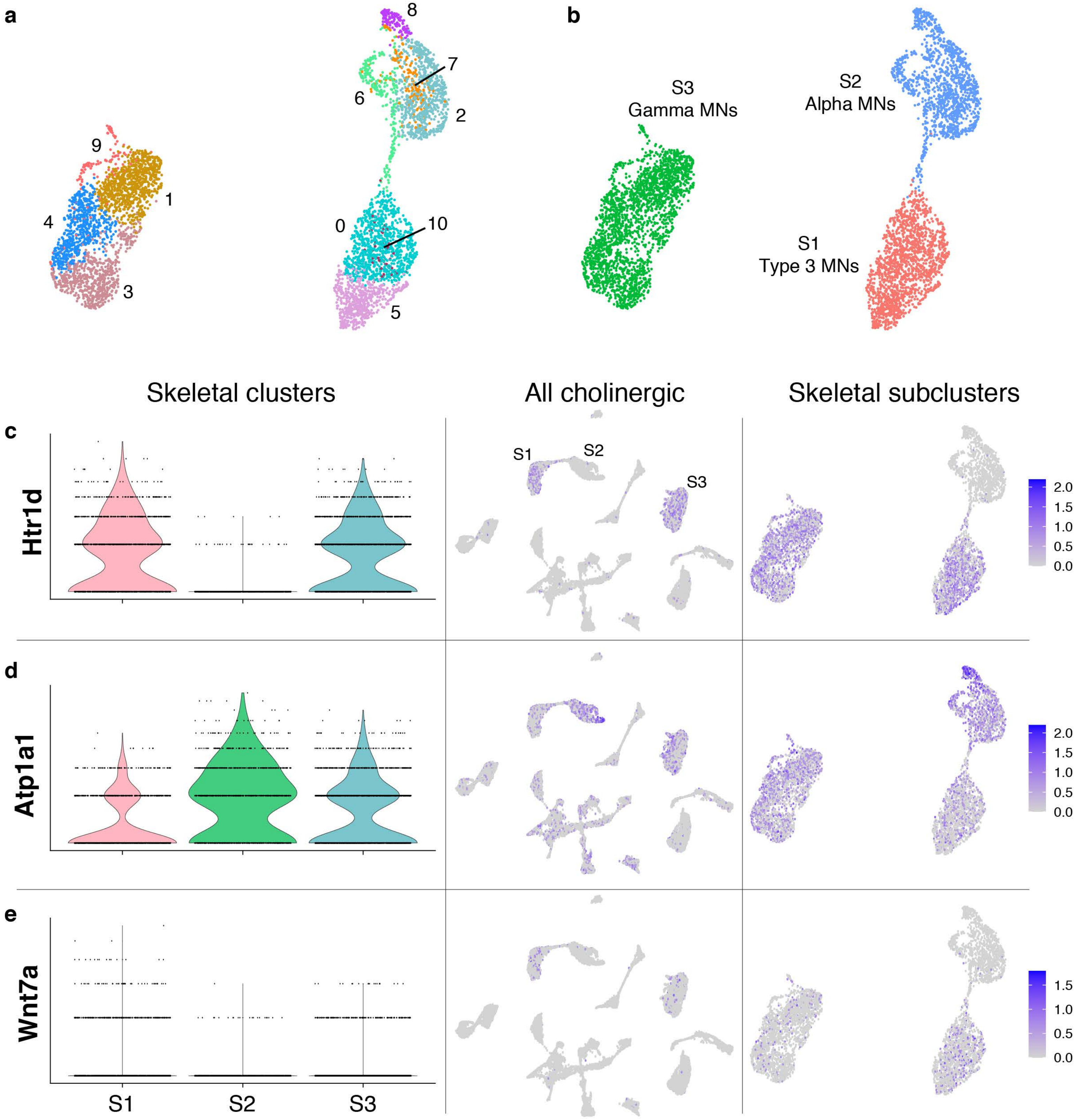

Supplementary Fig. 7. Expression of known skeletal subtype markers in our dataset. a, b, Subclustering of just the skeletal MNs reveals 4 subtypes of alpha, 3 of Type 3, and 4 of gamma MNs. **c,** The serotonin receptor 1D (*Htr1d*) has been described as a marker mainly expressed in gamma MNs, but is widely expressed in skeletal MNs in our nuclear sequencing dataset. **d,** The ATPase Na+/K+ transporting alpha 1 (*Atp1a1*) is considered a marker for alpha motor neurons, but is widely expressed in our dataset. **e,** *Wnt7a* is known to be expressed selectively in gamma MNs starting at late embryonic stages, but is found in both gamma and Type 3 MNs in our dataset.

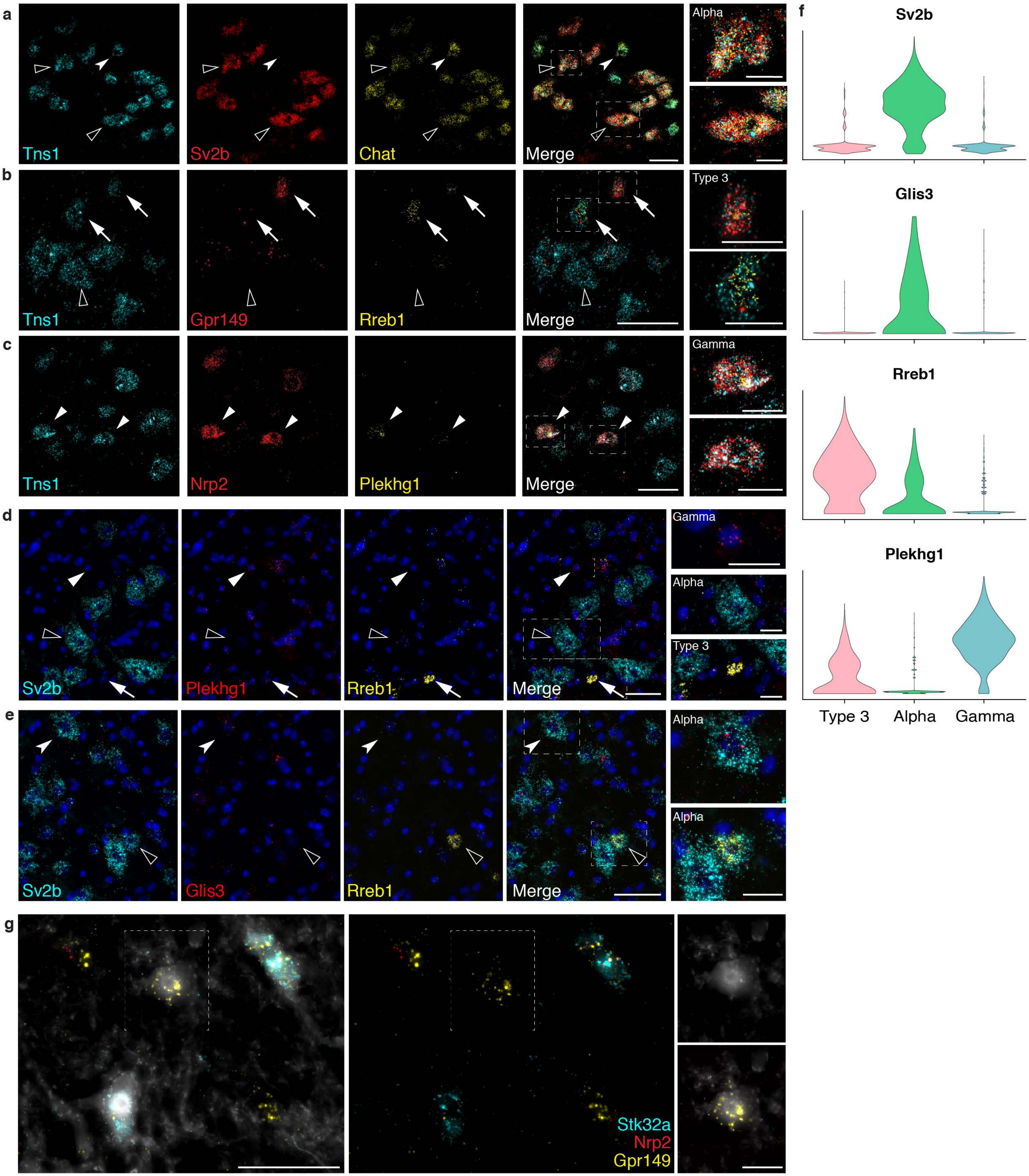

a
Tns1
Sv2b
Chat
Merge
Alpha
b
Tns1
Gpr149
Rreb1
Merge
Type 3
c
Tns1
Nrp2
Plekhg1
Merge
Gamma
d
Sv2b
Plekhg1
Rreb1
Merge
Gamma
Alpha
Type 3
e
Sv2b
Glis3
Rreb1
Merge
Alpha
Alpha
f
Sv2b
Glis3
Rreb1
Plekhg1
Type 3
Alpha
Gamma
g
Stk32a
Nrp2
Gpr149

Supplementary Fig 8. Expression of additional novel markers of alpha, gamma, and Type 3 MNs. **a,** ISH showing expression of novel alpha MN marker *Sv2b* (red) restricted to a subset of large diameter *Tns1*+ (cyan) *Chat*+ (yellow) neurons. Pointed arrowhead highlights example of a *Sv2b*-negative MN of smaller diameter (i.e. Type 3 or gamma). High magnification images of triple positive alpha MNs (open arrowheads). **b,** Smaller diameter Type 3 MNs among *Tns1*+ skeletal MNs (cyan) as revealed by expression of *Gpr149* (red) (arrow, top box), or *Rreb1* (yellow; arrow, bottom box) near a large diameter MN negative for both markers (open arrowhead). **c,** Field of *Tns1*+ skeletal MNs (cyan) containing four *Nrp2*-expressing gamma MNs (red), some of which co-expressed *Plekhg1* (yellow, filled arrowheads). Cells positive only for *Tns1* correspond skeletal MN types other than gamma. **d,** Lumbar spinal cord images show that *Plekhg1* alone represents gamma MNs (filled arrowhead, top), *Sv2b* (cyan) labels alpha MNs (open arrowhead, middle); *Rreb1* (yellow) alone or co-expressed with *Plekhg1* (red) represent Type 3 MNs (arrow, bottom). **e**, Sacral spinal cord images show that *Glis3* (red) expression is restricted to a subset of alpha MNs (*Sv2b*+, cyan) (pointed arrowhead, top). A subset of alpha MNs express *Rreb1* (yellow) (open arrowhead, bottom). **f,** Violin plots of *Sv2b*, *Glis3*, *Rreb1*, and *Plekhg1* expression in alpha, gamma, and Type 3 MNs. **g,** High magnification image from Fig. 3e of a Type 3 MN without C-boutons expressing *Gpr149.* Scale bars, 50 µm. High magnification scale bars, 20 µm.

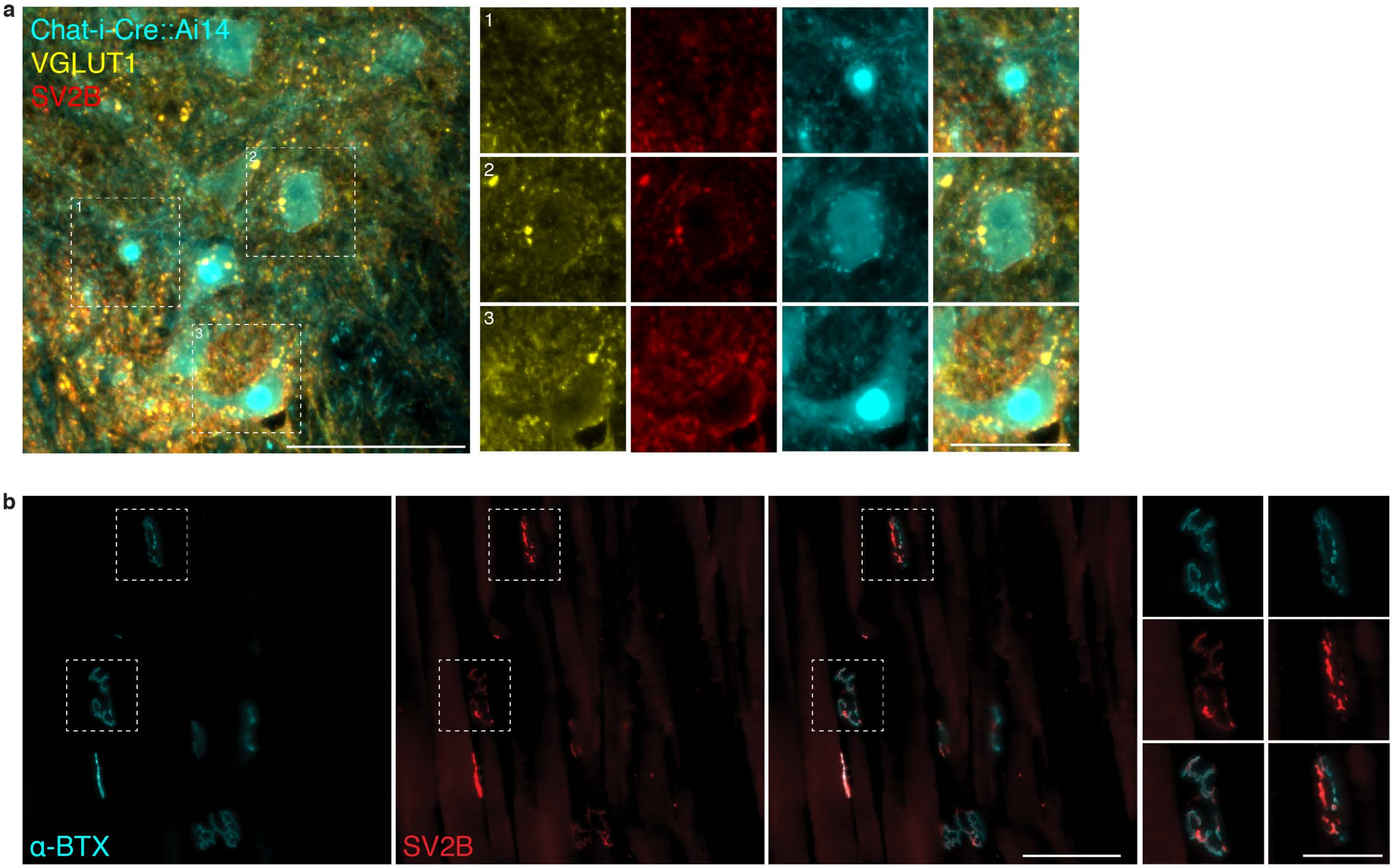

Supplementary Fig 9. Novel alpha MN marker SV2B is detected in the spinal cord and at the neuromuscular junction. a, Section of spinal cord ventral horn showing co-expression of VGLUT1 (yellow) and SV2B (red) in Chat-Cre::Ai14 labeled motor neurons (cyan). High magnifications of MN cell bodies showing presence of VGLUT1+ synapses (yellow) and C boutons (cyan) on SV2B+ alpha MNs (boxes 2 and 3) and lack of both synapse types on a SV2B- gamma or Type 3 MN (box 1). Interestingly, the VGLUT1 and SV2B signals appear localize to the same synapses; the overlapping yellow and red signals are not due to bleed through since they correspond to Alexa488 and Alexa647 respectively. **b,** Section of tibialis anterior muscle showing co-expression of SV2B (red) with the NMJ marker a-bungarotoxin (cyan). High magnification images highlight 2 NMJs co-expressing both markers. Low magnification scale bar, 100 µm. High magnification scale bar, 50 µm.

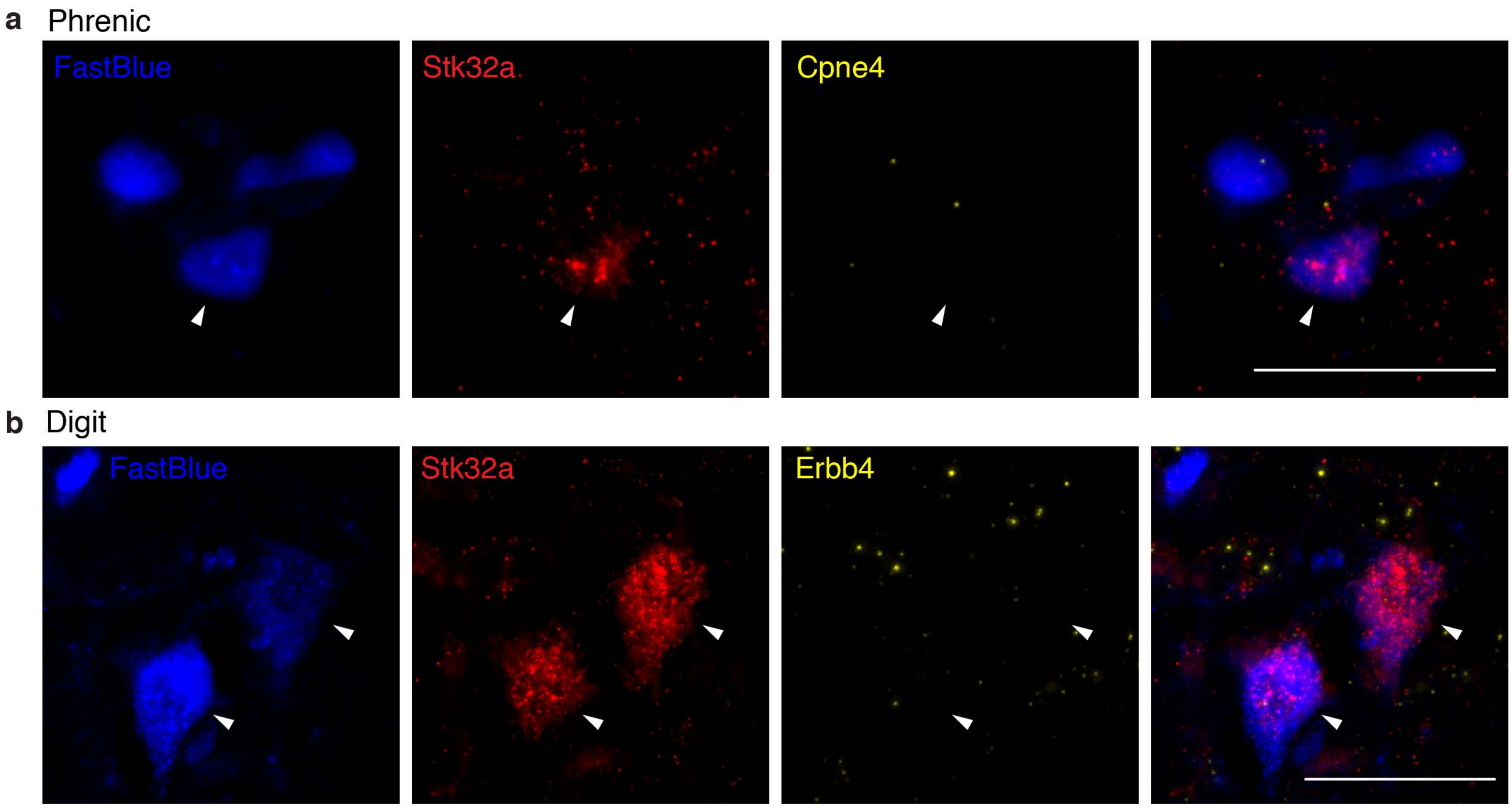

Supplementary Fig. 10. Additional examples of alpha motor neuron cluster marker expression in retrogradely labeled neurons. a, ISH of retrogradely labeled tissue showing no expression of the cluster 4 digit-innervating MN marker *Cpne4* (yellow) in phrenic MNs (blue). Filled arrowhead highlights a phrenic MN expressing high levels of *Stk32a* that was included in the analysis for alpha MNs. **b,** ISH of retrogradely labeled tissue showing lack of expression of the cluster 7 phrenic MN marker *Erbb4* (yellow) in digit-innervating MNs (blue). Scale bars, 50 µm.

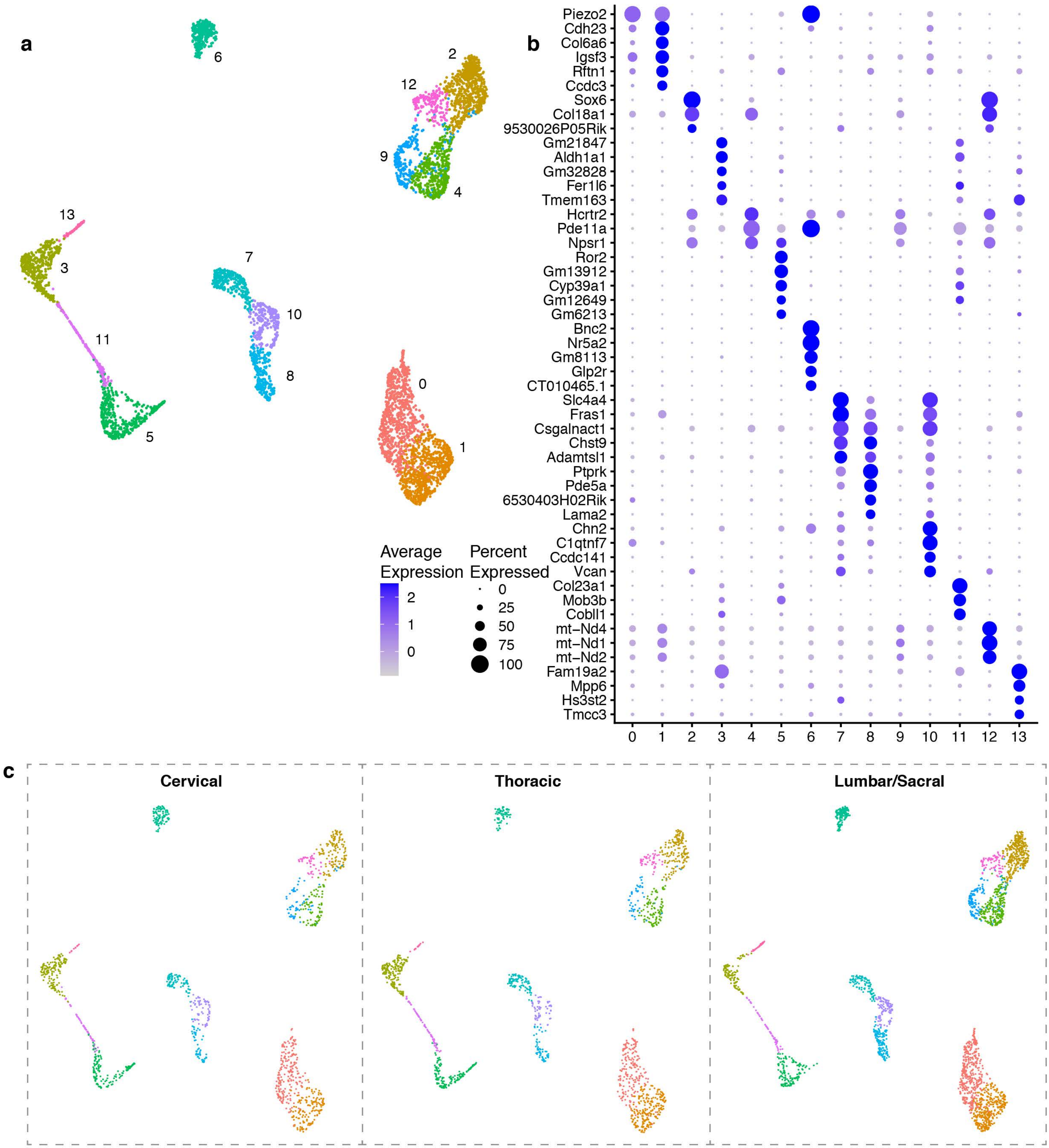

Supplementary Fig. 11. Subclustering of cholinergic interneurons reveals 14 subtypes. a, Cholinergic interneurons can be subclustered into 14 distinct subtypes. **b,** Dot plot showing expression of the top 5 marker genes for each interneuron subcluster. **c,** UMAPs of cholinergic interneurons detected in this study at each spinal cord level.

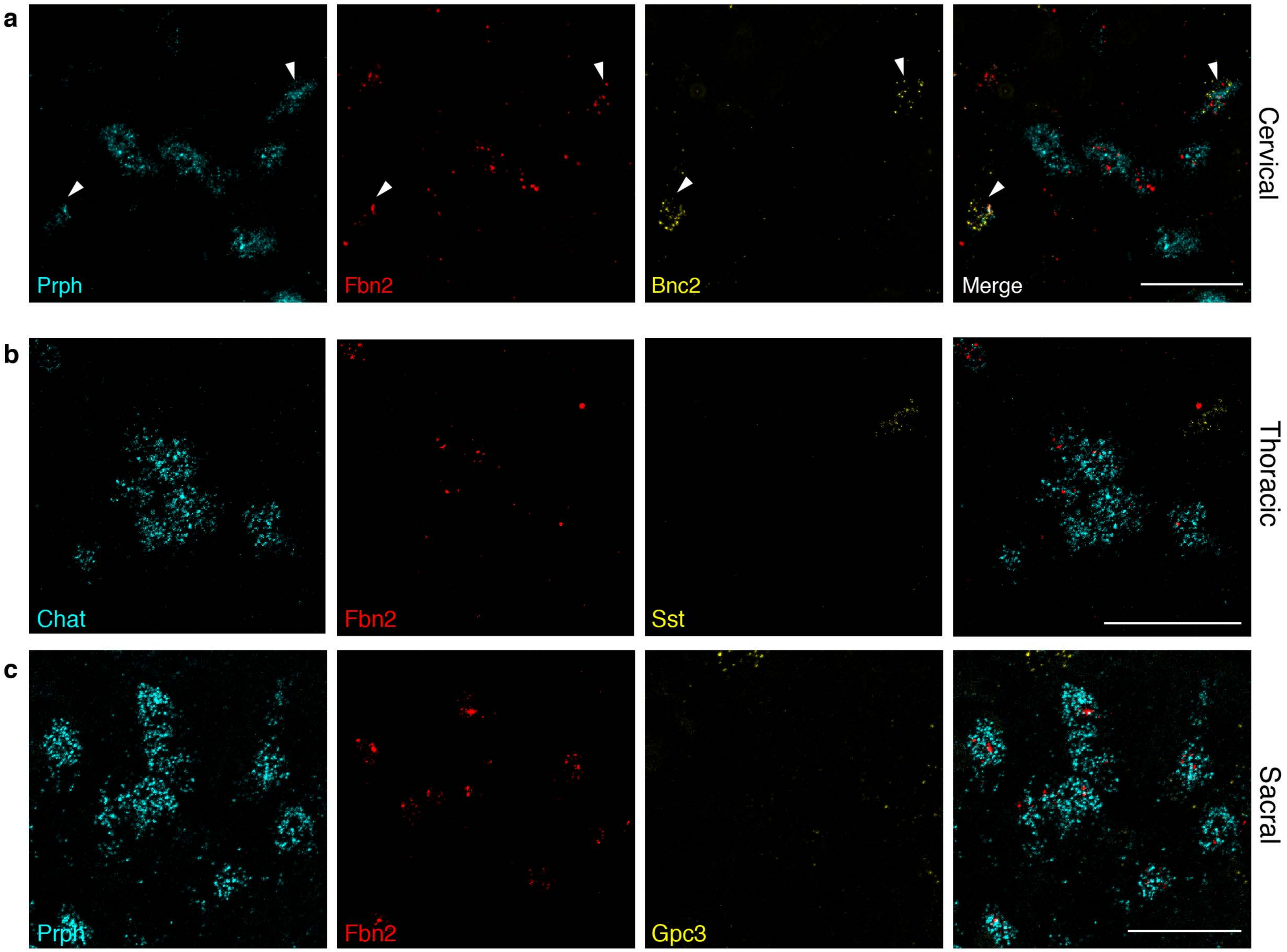

Supplementary Fig. 12. Markers for subtypes of visceral MNs in different spinal cord levels. a, *Bnc2* labels a small population of visceral MNs only in the cervical spinal cord. Arrowheads highlight cells that coexpress *Bnc2* (yellow), the visceral MN marker *Fbn2* (red), and the MN marker *Prph* (cyan). **b,** ISH highlighting visceral MNs negative for sacral-specific cluster marker *Sst* (yellow) in thoracic spinal cord. **c,** ISH highlighting visceral MNs negative for cervical-specific cluster marker *Gpc3* (yellow) in sacral spinal cord. Scale bars, 50 µm.

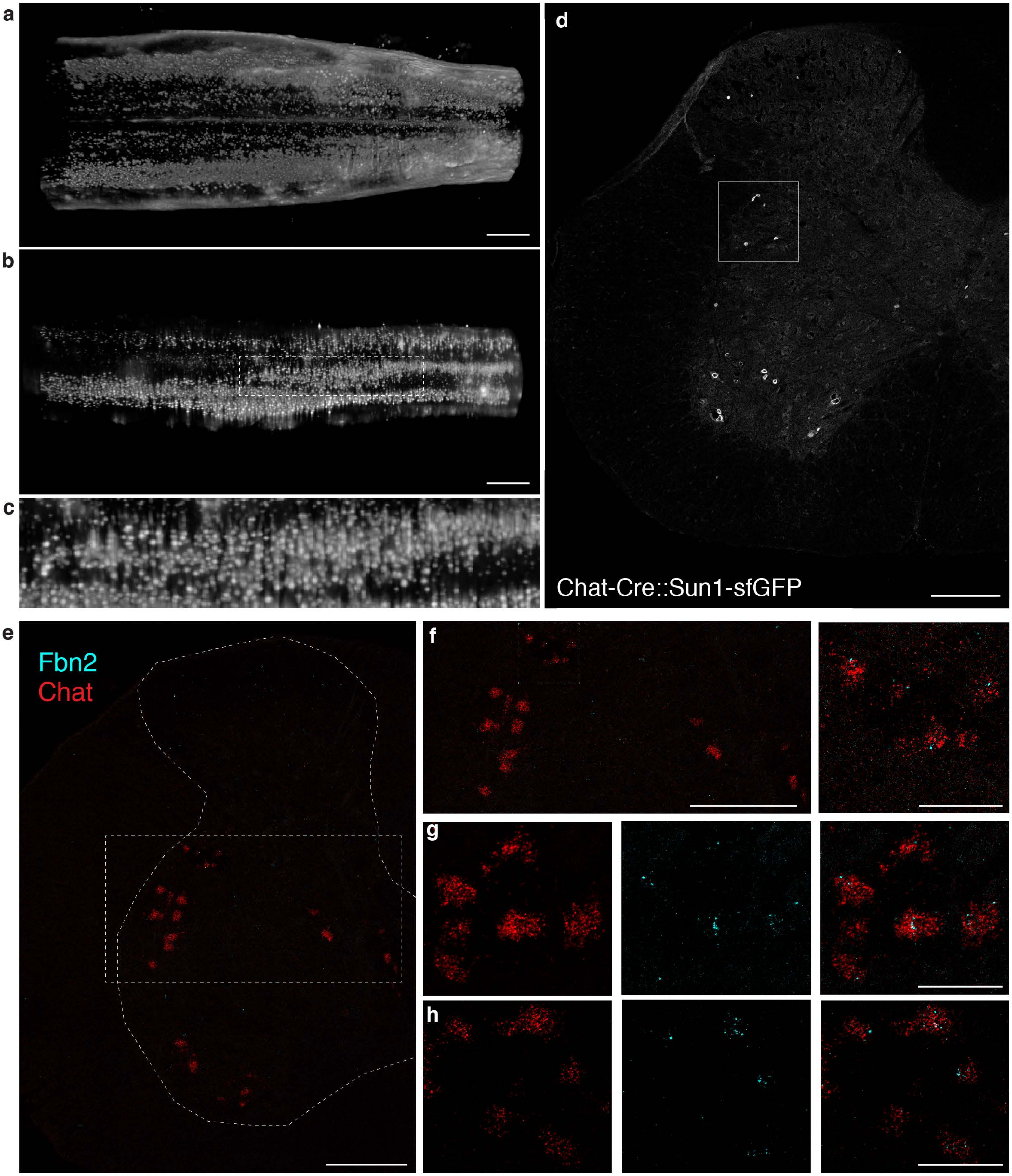
a
b
c
d
Chat-Cre::Sun1-sfGFP
e
Fbn2
Chat
f
g
h

Supplementary Fig. 13. The intermediolateral cell column, where visceral MNs are found, extends into the cervical spinal cord. a, Dorsal view of a whole, cleared cervical SC from a Chat-Cre::Sun1-sfGFP mouse showing GFP expression. **b,** Lateral view of a whole, cleared cervical SC, aligned with the dorsal view above (a), showing the extension of the intermediolateral cell column into lower levels of the cervical SC. **c,** High magnification view of the lateral column highlighted in b. **d,** Coronal view of Chat-Cre::Sun1-sfGFP in cervical spinal cord, showing nuclear envelope GFP expression in the lateral horn (boxed). **e,** ISH showing expression of *Fbn2* in *Chat*+ cervical SC neurons. **f,** Boxed region from e and higher magnification view of the lateral horn with distinct *Fbn2* expression in lateral horn neurons. **g, h,** Additional high magnification examples of cervical lateral horn neurons exhibiting frequent co-expression of *Fbn2* and *Chat*. Whole tissue scale bars, 500 µm. Low magnification scale bars, 200 µm. High magnification scale bars, 50 µm.

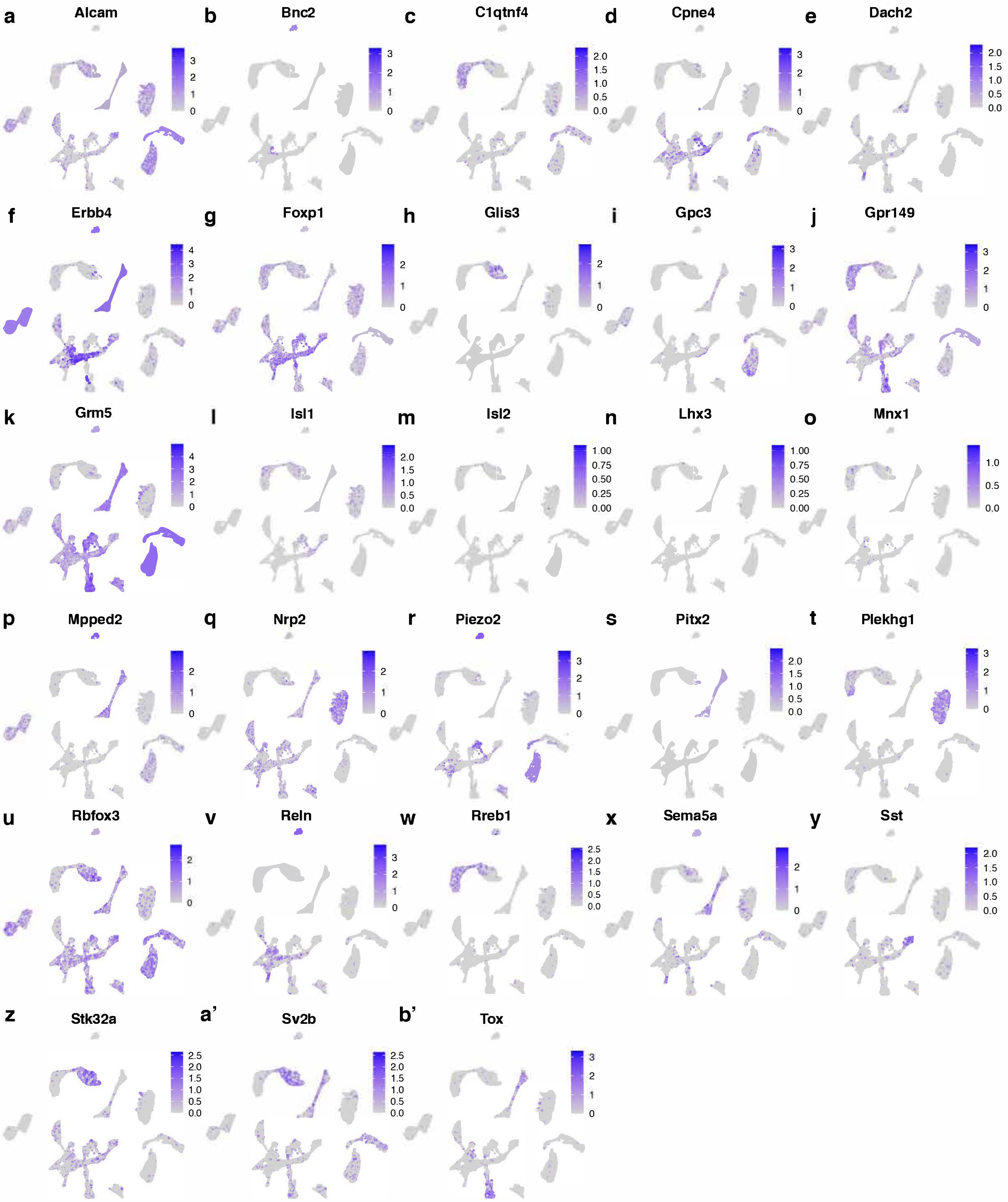

a Alcam
b Bnc2
c C1qtnf4
d Cpne4
e Dach2
f Erbb4
g Foxp1
h Glis3
i Gpc3
j Gpr149
k Grm5
l Isl1
m Isl2
n Lhx3
o Mnx1
p Mpped2
q Nrp2
r Piezo2
s Pitx2
t Plekhg1
u Rbfox3
v Reln
w Rreb1
x Sema5a
y Sst
z Stk32a
a’ Sv2b
b’ Tox

Supplementary Fig. 14. Nuclear expression across all cholinergic neurons sequenced for genes discussed in our analysis. a-b', UMAPs showing expression of genes probed by ISH across all cholinergic nuclei sequenced. Only displaying UMAPs not shown elsewhere. *Alcam*, *Bnc2*, *C1qtnf4*, *Cpne4*, *Dach2*, *Erbb4*, *Foxp1*, *Glis3*, *Gpc3*, *Gpr149*, *Grm5*, *Isl1*, *Isl2*, *Lhx3*, *Mnx1*, *Mpped2*, *Nrp2*, *Piezo2*, *Pitx2*, *Plekhg1*, *Rbfox3*, *Reln*, *Rreb1*, *Sema5a*, *Stk32a*, *Sst*, *Sv2b*, *Tox*.

Supplementary Table 1. Summary of number of nuclei sequenced in this study

All		All cholinergic		Skeletal		Alpha		Interneurons		Visceral	
Cluster	Count	Cluster	Count	Cluster	Count	Cluster	Count	Cluster	Count	Cluster	Count
0	2321	I1	1520	0	917	0	344	0	816	0	579
1	2243	I2	780	1	853	1	338	1	704	1	559
2	1914	I3	756	2	688	2	183	2	617	2	483
3	1737	I4	702	3	635	3	139	3	434	3	472
4	1598	I5	584	4	626	4	119	4	418	4	468
5	1577	I6	544	5	562	5	78	5	417	5	466
6	1563	I7	160	6	276	6	45	6	329	6	380
7	1513	I8	329	7	184	7	38	7	288	7	369
8	1458	V1	798	8	136			8	262	8	362
9	1304	V2	771	9	129			9	259	9	344
10	1235	V3	722	10	48			10	230	10	330
11	1226	V4	662					11	197	11	256
12	1213	V5	618					12	164	12	242
13	1213	V6	582					13	80	13	190
14	1019	V7	557							14	78
15	942	V8	467							15	35
16	929	V9	247								
17	795	V10	189								
18	673	S1	1528								
19	656	S2	1285								
20	604	S3	2241								
21	592										
22	578										
23	550										
24	539										
25	538										
26	532										
27	503										
28	487										
29	484										
30	334										
31	329										
32	270										
33	260										
34	250										
35	102										
36	98										
37	52										
**TOTAL:**	34231		16042		5054		1284		5215		5613

Supplementary Table 2. In situ probe and reagent information

Designation	Source or reference	Identifier
RNAscope® Fluorescent Multiplex Detection Reagents	Advanced Cell Diagnostics	ACD: 320851
RNAscope® 4-Plex Ancillary Kit for Multiplex Fluorescent Kit v2	Advanced Cell Diagnostics	ACD: 323120
RNAscope® Multiplex Fluorescent Reagent Kit v2	Advanced Cell Diagnostics	ACD: 323100
RNAscope® Probe-Mm-Bnc2-C2	Advanced Cell Diagnostics	ACD: 518521
RNAscope® Probe-Mm-C1qtnf4-C3	Advanced Cell Diagnostics	ACD: 892741
RNAscope® Probe-Mm-Chat-C2, C3	Advanced Cell Diagnostics	ACD: 410071
RNAscope® Probe-Mm-Cpne4-C1	Advanced Cell Diagnostics	ACD: 474721
RNAscope® Probe-Mm-Dach2-C1	Advanced Cell Diagnostics	ACD: 839361
RNAscope® Probe-Mm-Erbb4-C3	Advanced Cell Diagnostics	ACD: 318721
RNAscope® Probe-Mm-Fbn2-C1, C2	Advanced Cell Diagnostics	ACD: 313881
RNAscope® Probe-Mm-Glis3-C1	Advanced Cell Diagnostics	ACD: 314161
RNAscope® Probe-Mm-Gpc3-C1	Advanced Cell Diagnostics	ACD: 418541
RNAscope® Probe-Mm-Gpr149-C1	Advanced Cell Diagnostics	ACD: 318071
RNAscope® Probe-Mm-Grm5-C2	Advanced Cell Diagnostics	ACD: 423631
RNAscope® Probe-Mm-Gulp1-C1	Advanced Cell Diagnostics	ACD: 490061
RNAscope® Probe-Mm-Mpped2-C1	Advanced Cell Diagnostics	ACD: 848371
RNAscope® Probe-Mm-Nrp2-C1	Advanced Cell Diagnostics	ACD: 500661
RNAscope® Probe-Mm-Piezo2-C3	Advanced Cell Diagnostics	ACD: 400191
RNAscope® Probe-Mm-Pitx2-C2	Advanced Cell Diagnostics	ACD: 412841
RNAscope® Probe-Mm-Plekhg1-C1, C2	Advanced Cell Diagnostics	ACD: 411501
RNAscope® Probe-Mm-Prph-C2, C3	Advanced Cell Diagnostics	ACD: 400361
RNAscope® Probe-Mm-Rbfox3-C1	Advanced Cell Diagnostics	ACD: 313311
RNAscope® Probe-Mm-Reln-C1	Advanced Cell Diagnostics	ACD: 405981
RNAscope® Probe-Mm-Rreb1-C2	Advanced Cell Diagnostics	ACD: 542641
RNAscope® Probe-Mm-Sema5a-C3	Advanced Cell Diagnostics	ACD: 508091
RNAscope® Probe-Mm-Slc6a1-C3	Advanced Cell Diagnostics	ACD: 444071
RNAscope® Probe-Mm-Sst-C2, C3	Advanced Cell Diagnostics	ACD: 404631
RNAscope® Probe-Mm-Stk32a-C4	Advanced Cell Diagnostics	ACD: 509691
RNAscope® Probe-Mm-Sv2b-C3	Advanced Cell Diagnostics	ACD: 479991
RNAscope® Probe-Mm-Tns1-C1, C3	Advanced Cell Diagnostics	ACD: 551411
RNAscope® Probe-Mm-Tox-C1	Advanced Cell Diagnostics	ACD: 484781
RNAscope® Probe-Mm-Zeb2-C2	Advanced Cell Diagnostics	ACD: 436391
Pyranose Oxidase	Sigma-Aldrich	Sigma: P4234
SSC Buffer, 20X Concentrate	Sigma-Aldrich	Sigma: SRE0068-1L
RNase Inhibitor, Murine	New England BioLabs	NEB: M0314L
Opal 520 Reagent Pack	Akoya Biosciences	Akoya: FP1487001KT
Opal 570 Reagent Pack	Akoya Biosciences	Akoya: FP1488001KT
Opal 620 Reagent Pack	Akoya Biosciences	Akoya: FP1495001KT
Opal 690 Reagent Pack	Akoya Biosciences	Akoya: FP1497001KT
