# Supplementary material for: Single nucleus RNA-sequencing defines unexpected diversity of cholinergic neuron types in the adult mouse spinal cord
Source: Nat Commun. 2021 Apr 30;12:2471. doi: 10.1038/s41467-021-22691-2 (PMC8087807; doi:10.1038/s41467-021-22691-2)
Supplement: Supplementary file 3 — Description of Additional Supplementary Files [file 41467_2021_22691_MOESM3_ESM.pdf]

Supplementary Data 1. All markers differentiating between cell types identified in this study.

Supplementary Data 2. Activity-related alpha MN marker genes.

Supplementary Movie 1. Whole tissue clearing of Chat-IRES-Cre; Sun1-sfGFP lumbar spinal cord with immunolabeling of nuclei sequenced in this study.

Lumbar spinal cord sample processed by iDisco using anti-GFP antibodies to detect Sun1-positive nuclei of cholinergic neurons.

Supplementary Movie 2. Whole tissue clearing of Chat-IRES-Cre; Sun1-sfGFP cervical spinal cord with immunolabeling of nuclei sequenced in this study.

Cervical spinal cord region processed by iDisco using anti-GFP antibodies to detect Sun1-positive nuclei of cholinergic neurons.
