# Supplementary material for: Single nucleus RNA-sequencing defines unexpected diversity of cholinergic neuron types in the adult mouse spinal cord
Source: Nat Commun. 2021 Apr 30;12:2471. doi: 10.1038/s41467-021-22691-2 (PMC8087807; doi:10.1038/s41467-021-22691-2)
Supplement: Supplementary file 8 — Reporting Summary [file 41467_2021_22691_MOESM8_ESM.pdf]

nature research

Corresponding author(s): Claire Le Pichon

Last updated by author(s): Feb 27, 2021

Reporting Summary

Nature Research wishes to improve the reproducibility of the work that we publish. This form provides structure for consistency and transparency in reporting. For further information on Nature Research policies, see our Editorial Policies and the Editorial Policy Checklist.

Statistics

For all statistical analyses, confirm that the following items are present in the figure legend, table legend, main text, or Methods section.

n/a	Confirmed	
[ ]	[x]	The exact sample size (*n*) for each experimental group/condition, given as a discrete number and unit of measurement
[ ]	[x]	A statement on whether measurements were taken from distinct samples or whether the same sample was measured repeatedly
[ ]	[x]	The statistical test(s) used AND whether they are one- or two-sided<br>*Only common tests should be described solely by name; describe more complex techniques in the Methods section.*
[x]	[ ]	A description of all covariates tested
[ ]	[x]	A description of any assumptions or corrections, such as tests of normality and adjustment for multiple comparisons
[ ]	[x]	A full description of the statistical parameters including central tendency (e.g. means) or other basic estimates (e.g. regression coefficient) AND variation (e.g. standard deviation) or associated estimates of uncertainty (e.g. confidence intervals)
[ ]	[x]	For null hypothesis testing, the test statistic (e.g. *F*, *t*, *r*) with confidence intervals, effect sizes, degrees of freedom and *P* value noted<br>*Give P values as exact values whenever suitable.*
[x]	[ ]	For Bayesian analysis, information on the choice of priors and Markov chain Monte Carlo settings
[x]	[ ]	For hierarchical and complex designs, identification of the appropriate level for tests and full reporting of outcomes
[x]	[ ]	Estimates of effect sizes (e.g. Cohen's *d*, Pearson's *r*), indicating how they were calculated

Our web collection on statistics for biologists contains articles on many of the points above.

Software and code

Policy information about availability of computer code

Data collection	Cell Ranger v3.1.0, 10X Genomics, Zeiss Zen Software (blue edition), FIJI v2.0.0-rc-69/1.52p
Data analysis	Seurat package 3.1.4, R Studio v1.1.463, GraphPad Prism v9.0.0

For manuscripts utilizing custom algorithms or software that are central to the research but not yet described in published literature, software must be made available to editors and reviewers. We strongly encourage code deposition in a community repository (e.g. GitHub). See the Nature Research guidelines for submitting code & software for further information.

Data

Policy information about availability of data

All manuscripts must include a data availability statement. This statement should provide the following information, where applicable:
- Accession codes, unique identifiers, or web links for publicly available datasets
- A list of figures that have associated raw data
- A description of any restrictions on data availability

The datasets generated during and/or analyzed during the current study are available on GEO (accession # GSE167597) and at spinalcordatlas.org.

Field-specific reporting

Life sciences study design

All studies must disclose on these points even when the disclosure is negative.

Sample size	No statistical methods were used to predetermine sample size for nuclear sequencing. Pooled sample size (n=12 animals) was deemed sufficient based on the number of single nuclei obtained after quality control (>6000 single nuclei), with appropriate detection of multiple and distinct cell-type clusters. Furthermore, analyzing just 1 of 3 replicates of sequenced nuclei did not dramatically change the clustering pattern nor the major conclusions. For quantitative validation experiments by in situ hybridization, we predetermined sample size prior to image collection, without sample size calculation.
Data exclusions	Sequencing data were filtered according to standard Seurat analysis criteria: outliers were identified based on number of expressed genes and mitochondrial proportions and removed from the data. Neurons labeled by in situ hybridization were excluded from quantitative analyses if, under blinded conditions, the number of Stk32a- or Fbn2-positive RNA puncta was indistinguishable from background labeling.
Replication	Single nucleus data were replicated 3 times, and resulted in consistent clustering patterns and major conclusions. Sequencing data were validated by multiplexed fluorescent in situ hybridization (ISH) in multiple probe combinations, across at least 5 sections from multiple animals. All attempts to validate data by ISH as predicted by the sequencing were successful.
Randomization	Randomization is not relevant to our study since all samples were processed in parallel and pooled prior to sequencing and analysis.
Blinding	Clustering of single nuclei was performed in an unbiased and blinded manner, using standard programming code. Cluster names were assigned after generation of clusters. Image quantification was performed by blinded observers. Quantifications were all performed blind to sample identity.

Reporting for specific materials, systems and methods

We require information from authors about some types of materials, experimental systems and methods used in many studies. Here, indicate whether each material, system or method listed is relevant to your study. If you are not sure if a list item applies to your research, read the appropriate section before selecting a response.

Materials & experimental systems

n/a	Involved in the study	
☐	☒	Antibodies
☒	☐	Eukaryotic cell lines
☒	☐	Palaeontology and archaeology
☐	☒	Animals and other organisms
☒	☐	Human research participants
☒	☐	Clinical data
☒	☐	Dual use research of concern

Methods

n/a	Involved in the study	
☒	☐	ChIP-seq
☐	☒	Flow cytometry
☒	☐	MRI-based neuroimaging

Antibodies

Antibodies used	anti-SV2B (Synaptic Systems# 119 102, 1:500), anti-VGLUT1 (Synaptic Systems #135 011, 1:500)
Validation	Both these antibodies were validated by the manufacturer as specific using knockout mouse tissue.<br>anti-SV2B https://sysy.com/product/119102, PubMed: 19381277<br>anti-VGLUT1 https://sysy.com/product/135011#gallery-1

Animals and other organisms

Policy information about studies involving animals; ARRIVE guidelines recommended for reporting animal research

Laboratory animals	Male and female mice; Chat-IRES-Cre line crossed to CAG-sfGFP/Sun1 reporter on the C57Bl/6J background; 8 weeks of age. Housing conditions: 12/12 hour light/dark cycle, humidity between 30-50%, temperature 72ºC.
Wild animals	The study did not involve wild animals.
Field-collected samples	The study did not involve samples collected in the field.
Ethics oversight	All experiments involving animals were performed in accordance with animal protocol 17-003 and 20-003 approved by the NICHD ACUC.

Note that full information on the approval of the study protocol must also be provided in the manuscript.

Flow Cytometry

Plots

Confirm that:

- [x] The axis labels state the marker and fluorochrome used (e.g. CD4-FITC).
- [x] The axis scales are clearly visible. Include numbers along axes only for bottom left plot of group (a 'group' is an analysis of identical markers).
- [x] All plots are contour plots with outliers or pseudocolor plots.
- [x] A numerical value for number of cells or percentage (with statistics) is provided.

Methodology

Sample preparation	Nuclei were harvested and purified from frozen adult mouse spinal cord as described in the 'Methods' section. Nuclei were sorted directly into 1.5 ml eppendorf tubes containing 10ul solution for direct processing with the 10X Genomics Single Cell Controller.
Instrument	Sony Biotechnology SH800S Cell Sorter, Model LE-SH800SG
Software	Standard software package using CoreFinder that comes with all Sony SH800 sorters, data file structure using Flow Cytometry Standard (FCS) 3.1.
Cell population abundance	~0.8% of all events and ~1.1% of all singlet DRAQ5+ nuclei were EGFP-positive. See Extended Data Figure 1 for a complete breakdown of the gated events and percentages. For initial samples, GFP and Draq5 expression was confirmed under the microscope.
Gating strategy	As outlined in Extended Data Figure 1, Forward Scatter Area (FSC-A) vs. Back Scatter Area (BSC-A) was first used to gate nuclei from debris. Then this population was further purified by gating for Draq5-positive nuclei (FSC-Area vs. DRAQ5-Area) and ensuring singlet nuclei (DRAQ5-Area vs. DRAQ5-Height). These single nuclei were then gated into GFP-negative and GFP-positive populations (EGFP-Area vs. FSC-Area).

- [x] Tick this box to confirm that a figure exemplifying the gating strategy is provided in the Supplementary Information.
